# Biguanide is a modifiable pharmacophore for recruitment of endogenous Zn^2+^ to inhibit cysteinyl cathepsins: review and implications

**DOI:** 10.1007/s10534-019-00197-1

**Published:** 2019-05-01

**Authors:** Thomas D. Lockwood

**Affiliations:** Departmant of Pharmacology, School of Medicine, Wright State University, Dayton, OH 45435 USA

**Keywords:** Cysteinyl proteases, Cysteinyl protease inhibitors, Zn^2**+**^, Biguanide pharmacophore, Benzoyl arginine amide substrate

## Abstract

**Abstract:**

Excessive activities of cysteinyl cathepsins (CysCts) contribute to the progress of many diseases; however, therapeutic inhibition has been problematic. Zn^2**+**^ is a natural inhibitor of proteases with CysHis dyads or CysHis(Xaa) triads. Biguanide forms bidentate metal complexes through the two imino nitrogens. Here, it is discussed that phenformin (phenylethyl biguanide) is a model for recruitment of endogenous Zn^2**+**^ to inhibit CysHis/CysHis(X) peptidolysis. Phenformin is a Zn^2**+**^-interactive, anti-proteolytic agent in bioassay of living tissue. Benzoyl-l-arginine amide (BAA) is a classical substrate of papain-like proteases; the amide bond is scissile. In this review, the structures of BAA and the phenformin-Zn^2**+**^ complex were compared in silico. Their chemistry and dimensions are discussed in light of the active sites of papain-like proteases. The phenyl moieties of both structures bind to the “S2” substrate-binding site that is typical of many proteases. When the phenyl moiety of BAA binds to S2, then the scissile amide bond is directed to the position of the thiolate-imidazolium ion pair, and is then hydrolyzed. However, when the phenyl moiety of phenformin binds to S2, then the coordinated Zn^2**+**^ is directed to the identical position; and catalysis is inhibited. Phenformin stabilizes a “Zn^2**+**^ sandwich” between the drug and protease active site. Hundreds of biguanide derivatives have been synthesized at the 1 and 5 nitrogen positions; many more are conceivable. Various substituent moieties can register with various arrays of substrate-binding sites so as to align coordinated Zn^2**+**^ with catalytic partners of diverse proteases. Biguanide is identified here as a modifiable pharmacophore for synthesis of therapeutic CysCt inhibitors with a wide range of potencies and specificities.

**Graphical abstract:**

Phenformin-Zn^2+^ Complex
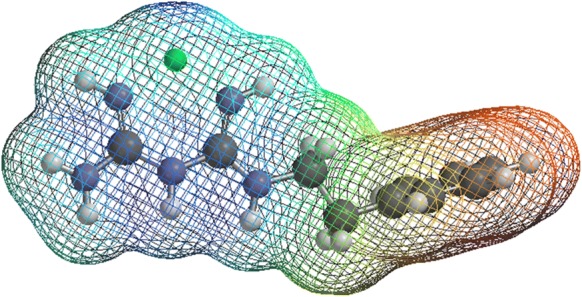

## Introduction: pharmacological recruitment of endogenous Zn^2+^ to decrease the reaction rates of cathepsins with CysHis dyads and CysHis(X) triads

The human genome encodes 11 cysteinyl cathepsins (CysCts) and multiple peptide inhibitors (Turk et al. 2012). CysCts serve general housekeeping functions in all cell types, and specialized roles in differentiated cells. Most have broad substrate acceptance and partially redundant functions. The early view was that cathepsins act only within the catabolic vacuole. However, it is now known that some CysCts can function in the extra-lysosomal and extracellular spaces until inactivated by oxidation or Nature’s inhibitors. Moreover, intra-vacuolar reaction rates can vary markedly in response to endogenous reaction conditions.

Despite mammalian complexity, deletion of the gene for an enzyme can be compared to “whole-body” inhibition of that enzyme. Various disease models have been compared in wild-type mice and those with deletions of genes for one or more CysCts (see below section). Deletions of various CysCt genes can decrease the progress of diverse pathogenic processes. Conversely, loss of endogenous protease inhibitors can increase these pathogenic processes. Optimal health and longevity require an unexplained balance between CysCt activities vs. Nature’s anti-proteolytic agents. Pharmacological opposition of one or more CysCt activity(s) might have great therapeutic benefits (Kramer et al. 2017).

Hydrolases referred to as “cysteinyl” proteases have catalytic dyads consisting of CysHis or triads of CysHis(X), where X is one of several amino acids, e.g. asparagine. Site-directed mutagenesis reveals that the essential catalytic partners are CysHis. The third amino acid of triads can be replaced with a non-catalytic amino acid without loss of activity; although this changes reaction kinetics (e.g. Vernet et al. 1995; Oh and Carringtion 1989). The mechanistic role of the third amino acid in triads is uncertain.

Over a century of investigations, biochemists have standardized CysHis/CysHis(X) protease assays with a reaction buffer consisting of metal chelator, sulfhydryl reducing agent and optimal pH. Standard assay conditions can buffer a constant maximal reaction rate. However, Nature varies the in vivo reactions of CysCts over a wide range by changing the composition of their endogenous reaction buffer. Machinery regulating metals, redox and protons at the cellular and compartmental levels has been identified. Metals, redox and protons are now recognized as simultaneous variables that modulate the endogenous CysHis/CysHis(X) reaction mechanism (Lockwood 2013).  

An exploitable feature of CysCts has been overlooked. All proteases with the CysHis/CysHis(X) catalytic partners share something in common: Zn^2**+**^ is a natural inhibitory modulator of their reaction mechanism. Zn^2**+**^ forms multi-dentate associations with the thiolate anion of cysteine neighboring the imidazole ring of histidine in many Zn^2**+**^ finger proteins, e.g. Cys_**2**_ -Zn^2**+**^-His_**2**_ (Kochanczyk et al. 2015; Kluska et al. 2018). The coordination properties of Zn^2**+**^ with Cys-His are flexible; various stoichiometries and stabilities are found: Cys_n_–Zn^2+^–His_n_. The bidentate interaction of Zn^2**+**^ with 1 Cys(thiolate) neighboring 1 His(imidazole) in catalytic sites is emerging as a major component of Nature’s reaction buffer. Recent evidence suggests that cell Zn^2**+**^ homeostasis is interactive with multiple pathways of cell protein degradation (see below section).

Most of the cell content of CysCts consists of inactive zymogens. Excessive ongoing proteolysis can lead to decompensated activation of pro-proteases, positive feedback, and necroptosis or apoptosis (see below section). Lesser excesses of intracellular or extracellular activities can be gradually injurious over many years. For some uses, complete inhibition of one or more CysCts might be required, e.g. inhibition of viral or parasite proteases. Importantly, excessive inhibition of various proteolytic pathways can be pathogenic under prolonged administration (Lu et al. 2016; Ketterer et al. 2017). Neuropathic accumulation of denatured proteins is a particular concern. For most purposes, safe inhibition implies a decline from excessive activity toward restoration of non-injurious “normal” activity.

**Fig. 1:**
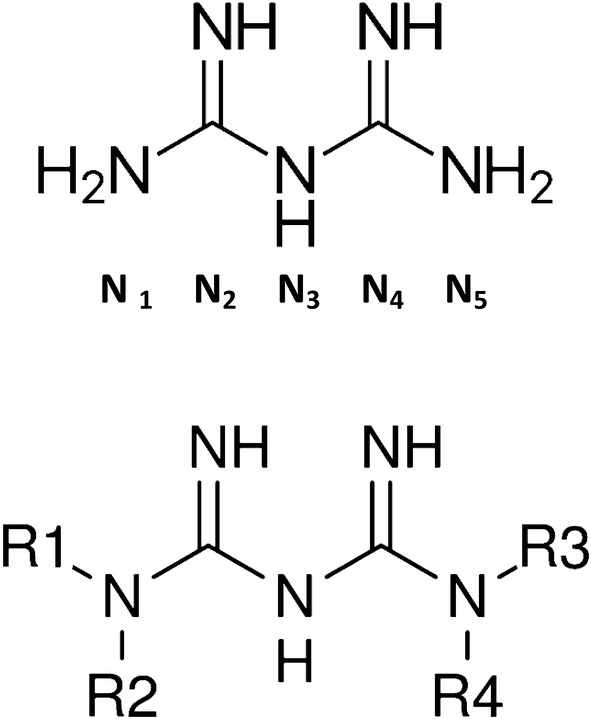
Top: structure of biguanide (guanyl guanidine). Bottom: substitution at the N-1 and N-5 positions of biguanide to produce many derivatives

**Fig. 2:**
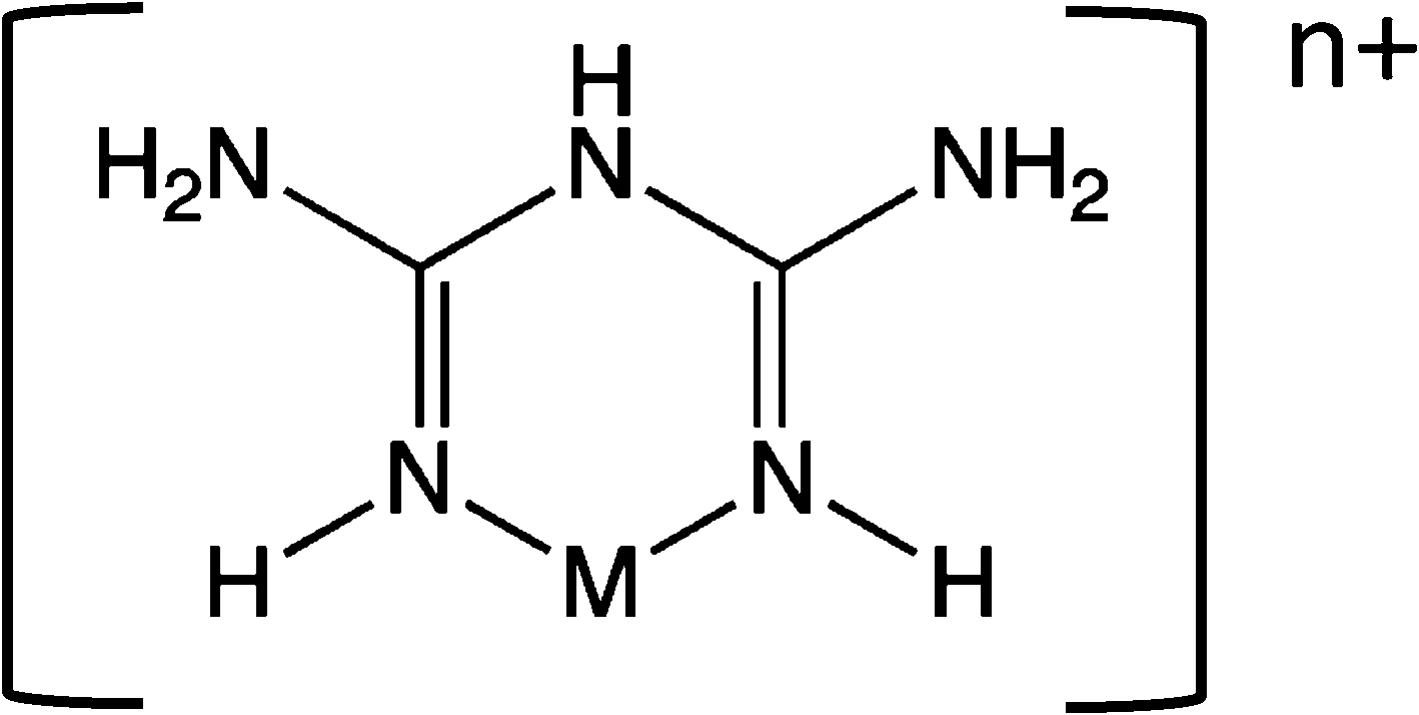
Structure of a 1:1 biguanide-metal complex through two imino nitrogens. The two lone electron pairs of imino nitrogens form bidentate coordination complexes with metal cations (see text). The charge on the complex is the charge of the metal cation. The structure shown can form mixed hetero-complexes with additional ligands coordinated around the central metal cation, e.g. a biguanide-Zn^2+^-protease “sandwich” as schematized in Fig. 6

If a drug could safely increase the anti-proteolytic effect of endogenous Zn^2**+**^, this might lower the excessive reaction rates of some or all CysCts. Biguanide (guanylguanidine) (Fig. 1) forms complexes with Zn^2**+**^ and other metal cations (Figs. 2, 4, 5). Certain biguanide derivatives have been found to be Zn^2**+**^-interactive inhibitors of protease reactions in enzyme assay and much of cell protein degradation in a perfused tissue bioassay (Sweeney et al. 2003; Lockwood 2010).

**Fig. 3:**
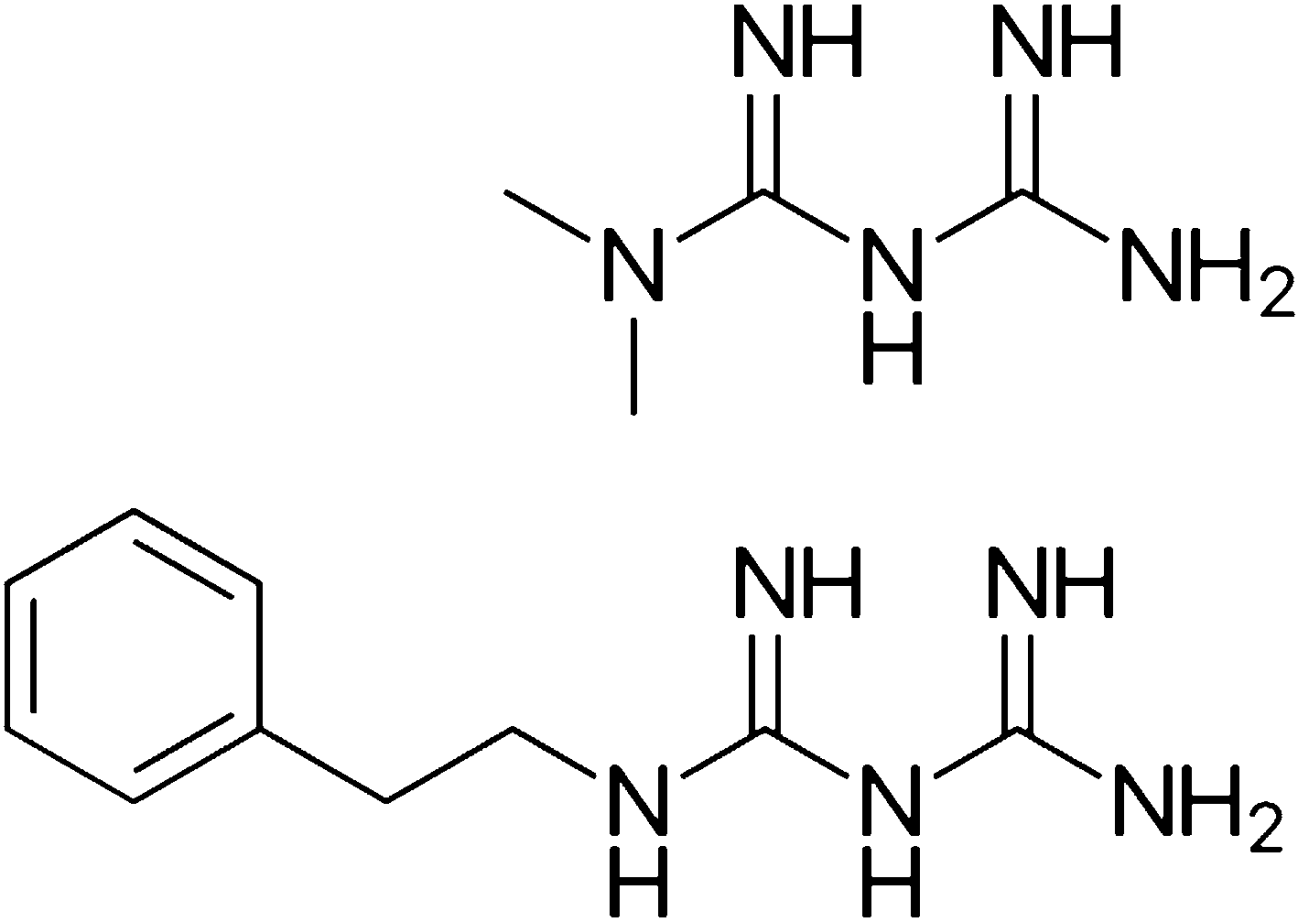
Structures of metformin and phenformin

In silico chemistry now permits precise comparison of small molecules. Phenformin is a derivative of biguanide (1-phenylethyl biguanide, Figs. 1, 2, 3). Benzoyl-l-arginine amide (BAA) is a well-known artificial substrate of diverse proteases. Comparison of the active site of papain-like proteases with the structures of BAA and the phenformin-Zn^2**+**^ complex explains why one is a substrate for proteases; and the other is an inhibitor. The Zn^2**+**^ complex of phenformin is a prototype for design or discovery of other anti-proteolytic biguanide derivatives with a wide range of specificities and potencies.

Present analysis involves two interactions of the protease active site with metal complexes of biguanide derivatives: (a) the interaction of CysHis/CysHis(X) catalytic partners with the coordinated metal cation and (b) the interaction of substrate-binding sites with derivative moieties of the biguanide pharmacophore (schematized in Figs. 5, 6).

## Much evidence suggests therapeutic benefits of cysteinyl cathepsin inhibition: a review of reviews

*Excessive function of one or more CysCts, or insufficiency of one or more endogenous inhibitors can contribute to multiple diseases and perhaps “normal” ageing*. In addition to intra-cellular injury, degradation of the extracellular matrix by CysCts can damage organ systems ranging from bone to vasculature (Korenč et al. 2015; Vizovišek et al. 2018). Moreover, CysCts such as cathepin C can act as intracellular convertases, activating co-compartmentalized zymogens of serine proteases that are released from inflammatory cells (see below section). Fortunately, excellent reviews survey recent progress; and repetition here is unnecessary. An incomplete sampling includes: (Reiser et al. 2010; Garsen et al. 2016; Ganesan 2017; Kramer et al. 2017; Taggart et al. 2017; Liu et al. 2018a; Korkmaz et al. 2018 and Lowry and Klegeris 2018).

A large body of literature implicates individual CysCts, groups of CysCts, or all CysCts in various neoplasias. However, their complex roles can lead to opposing effects on growth and metastasis (Anja et al. 2018; Pogorzelska et al. 2018). Much work remains to be done in the use of CysCt inhibitors against neoplasia.

Viruses, bacteria, fungi and parasites produce cysteinyl cathepsins with a variety of functions, thereby suggesting another potential use of inhibitors (e.g. Lindner et al. 2005; Sijwali and Rosenthal 2016; Verma et al. 2016; Agbowuro et al. 2018; Caffrey et al. 2018; McKerrow 2018). Host cell CysCts are also involved in the infectivity of some important viruses e.g. Ebola (Schornberg et al. 2006; Brix 2018).

*Cysteinyl cathepsin inhibitors can have “polytherapeutic” actions against low-grade inflammation combined with autoimmunity*. The combination of low-grade inflammation and autoimmunity contributes to diverse age-related diseases of separate causes. Multiple CysCts in various cell types underlie several processes required for autoimmunity and inflammation, e.g. lysosomal antigen processing, cytokine response, activation of serine protease zymogens, extracellular degradation, etc. Among many fine studies, a few milestones stand out for their clarity on the requirements of CysCts in inflammation e.g. (Guicciardi et al. 2001) and autoimmunity e.g. (Hsing et al. 2010).

It has been reviewed that mammalian inflammation and autoimmunity are increased by Zn^2**+**^ deficiency or dysregulation (Bonaventura et al. 2015; Gammoh and Rink 2017; Maywald et al. 2017; Wessels et al. 2017). A relationship between (a) Zn^2**+**^ dysregulation in various cell types and compartments, (b) excessive activity of one or more CysCts, and (c) elevated inflammatory/autoimmune processes seems likely. Such a relationship provides multiple points of therapeutic intervention.

## Pharmacological recruitment of endogenous Zn^2+^ to intervene in Cys-His catalysis

*Nature buffers the intracellular activities of CysHis/CysHis(X) cathepsins at submaximal rates by regulating components of their catalytic environment*. Positive and negative controls can be superimposed upon the buffered reaction. Redox regulation and metal regulation are virtually inseparable in the buffering of CysHis/CysHis(X) peptidolysis; and pH regulation can influence both. Many redox interactions can influence the status of protease sulfur, e.g. GSSG/GSH ratio or oxygenations (Lockwood 2002). A dedicated lysosomal reductase maintains reduction of disulfide bonds and activates CysHis/CysHis(X) cathepsins (see Rybicka et al. 2010; Balce et al. 2014; Allan and Yates 2015; Ewanchuk and Yates 2018). In addition to metal and redox regulation, the vacuolar proton pump (V-ATPase) is a major influence on the reaction rates of lysosomal CysCts, and the activation of pro-protease zymogens. Hypoxic acidified tissue promotes increased CysCt reaction rates. The relationship between V-ATPase action and lysosomal degradation is well known and omitted here (Xu and Ren 2015). Zn^2**+**^ can preemptively decrease the CysHis/CysHis(X) peptidolytic mechanism under otherwise optimal reaction conditions.

*The CysHis peptidolytic mechanism responds to the range of endogenous cell Zn*^*2***+**^*regulation*. Intracellular Zn^2**+**^ is 98% sequestered by protein binding. The biological concentration of truly “free” Zn^2**+**^ is very low, but not known with certainty (reviewed and discussed in Thompson and Fierke 2017). The concentration of free or aquo Zn^2**+**^, (i.e. with six hydrogen-bonded water molecules), has been suggested to be in the range of 1–100 picomolar. However, 1-3 micromolar cytoplasmic Zn^2**+**^ is neither free nor completely sequestered. Investigators have used various terms to refer to this intermediate form of Zn^2**+**^, e.g. “mobile”, “active”, “loosely buffered”, “exchangeable”, “chelatable” etc. These many forms of Zn^2**+**^ can be collectively referred to as “interactive” Zn^2**+**^. This Zn^2**+**^ exists in a dynamic exchange among myriad biomolecules with diverse Zn^2**+**^ affinities, e.g. citrate, phosphate, certain amino acids, peptides, and many more. Since Zn^2**+**^ has six ligand-binding sites (Cauët et al. 2010), occupancy of two or four sites, does not eliminate binding to a second ligand. Zn^2**+**^ interactions with various molecular structures and reactions need not be mediated exclusively by aquo Zn^2**+**^. In any case, pharmacological recruitment of endogenous Zn^2**+**^ to inhibit proteases does not require precise knowledge of the biological concentration of aquo Zn^2**+**^.

Long ago we studied the rate of bulk intracellular protein degradation in a viable perfused tissue bioassay, i.e. minute-to-minute release of incorporated 3H-leucine from cell proteins. Endogenous extracellular Zn^2**+**^ buffers were present in the perfusate. Much of tissue protein degradation was gradually inhibited by slight elevation of the extracellular Zn^2**+**^ concentration without injury (Sweeney et al. 2003; Lockwood 2010). In contrast, approximately 25% of bulk cell protein degradation was uninhibited by supra-physiological Zn^2**+**^ exposure indefinitely. We suggested that cell Zn^2**+**^ regulation and much of bulk protein degradation are inter-related. Subsequent evidence from many other groups is now supportive (see below section). Importantly, the lysosomal vacuole is a major participant in cell Zn^2**+**^ regulation as well as protein degradation.

The unique interaction of Zn^2**+**^ with the Cys(thiolate)-His(imidazole) catalytic partners is influenced by additional variables. CysHis/CysHis(X) peptidolysis can be inactivated by oxidation of protease sulfhydryl to diverse products (e.g. –SO, –SO_2_, –SO_3_, –S–S– etc.). Histidine can oxidize to 2-oxohistidine; however, the significance is uncertain. Many of the variables that interact with CysHis catalytic partners also interact with each other, independent of the protease. For example, glutathione interacts with CysHis proteolysis in several ways. The reducing action of GSH maintains reductive activation of protease sulfur. Oxidized GSSG inhibits cysteinyl proteases, thereby imparting sulfhydryl/disulfide redox buffering of the reaction (Lockwood 2002, Gong et al. 2018). In addition, the Zn^2**+**^-binding property of GSH also influences the “free” Zn^2**+**^ intensity (Helbig et al. 2008; Steiger et al. 2017).

## Growing evidence suggests that cell Zn^2+^ homeostasis influences diverse pathways of cell protein degradation and vice versa

*Zn*^2**+**^*-mediated anti-proteolytic mechanisms exist beyond the catalytic reaction*. Cell Zn^2**+**^ regulation has emerged as a modifier of diverse proteolytic processes. Functional classes of cysteinyl proteases include lysosomal proteases, calpains, caspases, and de-ubiquitinases. Interestingly, a ribosomal protein has unexplained cysteinyl protease activity of unknown function(s) (Sudhamalla et al. 2012). Zn^2**+**^ was found to be a particularly strong inhibitor of several caspases (e.g. Perry et al. 1997). Subsequently, it was discovered that Zn^2**+**^ inhibition of multiple caspases is mediated by Zn^2**+**^-binding allosteric exosites that are remote from their catalytic mechanisms (Eron et al. 2018). Certain viral proteases also have Zn^2**+**^ -binding exosites (reviewed in Parvez and Khan 2014). Calpain is inhibited by Zn^2**+**^ and activated by Zn^2**+**^ deficiency (Nakajima et al. 2014; Miyoshi et al. 2016). Zn^2**+**^ is known to interact with the E–F hand structure that is found on calpains and certain papain-like viral proteases. Although the 20S proteasome is a serine protease, it has been reported to have a Zn^2**+**^-binding site of high affinity; this binding site is presumably separate from the catalytic mechanism (Chouduri et al. 2008). Zn^2**+**^ reportedly induces inhibitory dissociation of the drosophila proteasome subunits (Kiss et al. 2005). Interestingly, most carboxyl proteases are largely uninhibited by the interactive cell Zn^2**+**^ intensity; and metallo-proteases require Zn^2**+**^ for activity.

*Zn*^2**+**^* is part of the regulation of lysosomal function; conversely lysosomes are a major part of cell Zn*^2**+**^*homeostasis*. Cell Zn^2**+**^ regulation includes ion channels, ATP-dependent metal transporters, proton-driven co-transporters, organic cation transporters (OCTs) and multidrug and toxicant transporters (MATEs) (reviewed in Hara et al. 2017; Fukada and Kambe 2018; Mikhaylina et al. 2018). Lysosomes have opposing Zn^2**+**^ import and export systems. Vacuolar Zn^2**+**^ transport serves at least three functions. (a) Lysosomes of some species can serve as Zn^2**+**^ storage depots for use in times of Zn^2**+**^ insufficiency (Roh et al. 2012). (b) Lysosomal Zn^2**+**^ regulation can influence lysosomal proteolytic function (Sweeney et al. 2003; Lockwood 2010). (c) Lysosomal uptake, sequestration and exocytosis of metals can defend the cytoplasm against metal overloading (Blaby-Haas and Merchant 2014; Kukic et al. 2014; Sharma et al. 2016a).

*Protein turnover can release protein-bound Zn*^2**+**^* during nutritional deficiency*. Nutritional availability of Zn^2**+**^ can limit the growth and metabolism of prokaryotes and humans. Since 98% of cell Zn^2**+**^ is sequestered within proteins, bulk protein degradation provides a large Zn^2**+**^ reservoir that can be released by increasing turnover. Zn^2**+**^ deficiency induces autophagy and releases sequestered Zn^2**+**^ from degraded cell proteins as investigated in yeast and plants. This restores interactive cell Zn^2**+**^ levels and promotes survival under Zn^2**+**^ deficiency as reported in several recent studies (Eguchi et al. 2017; Ding and Zhong 2017; Horie et al. 2017; Kawamata et al. 2017; Nakatogawa 2018). In theory, release of Zn^2**+**^ from degraded proteins can provide a self-limiting inhibitory effect or “brake” on the rate of Zn^2**+**^-sensitive protein turnover. In the future, Zn^2**+**^-interactive inhibitors of the CysHis catalytic mechanism might be combined with agents acting at separate sites on the same protease, or other sites of lysosomal and cellular Zn^2**+**^ regulation.

## Biguanide and biguanide derivatives form coordination complexes with Zn^2+^ and most other metal cations

**Fig. 4:**
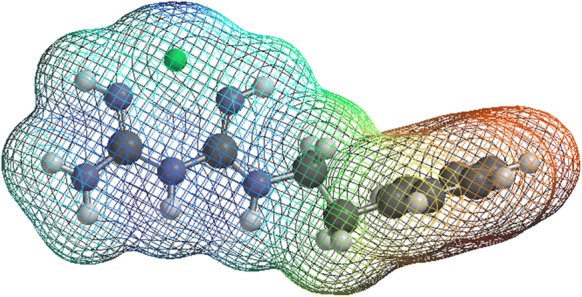
The phenformin-Zn^2+^ complex. The structure of the Zn^2+^ complex of phenformin was computed with the Spartan’08 program (Wavefunction, Inc., Irvine, CA) using density functional theory at the DFT B3LYP/6-31G* level. The computed structure is consistent with empirical observations under conditions and concentrations that are relevant to medicinal use (see text). Zn^2+^ forms a 1:1 biguanide complex through imino nitrogens at the 2 and 4 positions as illustrated in Fig. 2. Experimental evidence indicates that these nitrogens remain protonated as shown. In coordination complexes, electron density can be transferred from the ligand to the metal cation. The surface potential gradation from negative to positive is qualitatively indicated by gradation from red to blue in the mesh. Zn^2+^ is shown in green, carbon: black, nitrogen: blue, hydrogen: white. (Color figure online)

Guanidine is a Zn^2**+**^-binding ligand at neutral pH (Aoki et al. 2002). Biguanide is a bidentate metal ligand with much greater affinity for Zn^2**+**^ than guanidine. The lone electron pairs of the two imino nitrogens attract diverse metal cations, although affinities vary markedly (Prugnard and Noel 1996). Figure 4 illustrates the transfer of electron density from the bidentate biguanide ligand to the Zn^2**+**^ cation. Experimental evidence is consistent with this structure under pharmacological concentrations and conditions. With some metals, di-biguanide complexes can form with a central metal, i.e. 2 biguanides: 1 central metal. This depends upon the characteristics of the particular metal, and the concentrations or concentration ratios of ligand and metal.

The biguanide-Zn^2**+**^ formation constant (1/K_d_) is not precisely known; however, it is probably near 10 or 11 (i.e. K_d_ = 10^**−**11^). EDTA completely reverses the inhibitory action of Zn^2**+**^ and Zn^2**+**^-interactive biguanides. The K_d_ of the hexadentate Zn^2**+**^-EDTA complex is 10^**−**16.5^, corresponding to 10^5^–10^6^ times greater affinity than biguanides. Stabilities of biguanide complexes with various metals, and “on–off” kinetics can vary. The intracellular and extracellular distribution of biguanide among complexed endogenous metal species is unknown. The most effective anti-proteolytic metal complex need not be the complex that is formed in greatest amount.

## The history of a classical protease substrate: Benzoyl-l-arginine amide

In the late 1800s it was known that the papaya plant contains high amounts of a proteolytic activity (reviewed by Wurtz 1881). Papain became the namesake for a superfamily of proteases that are activated by reducing agents and inactivated by metal cations or oxidation of sulfhydryls (Rawlings 2017; Liu et al. 2018b).

**Fig. 5:**
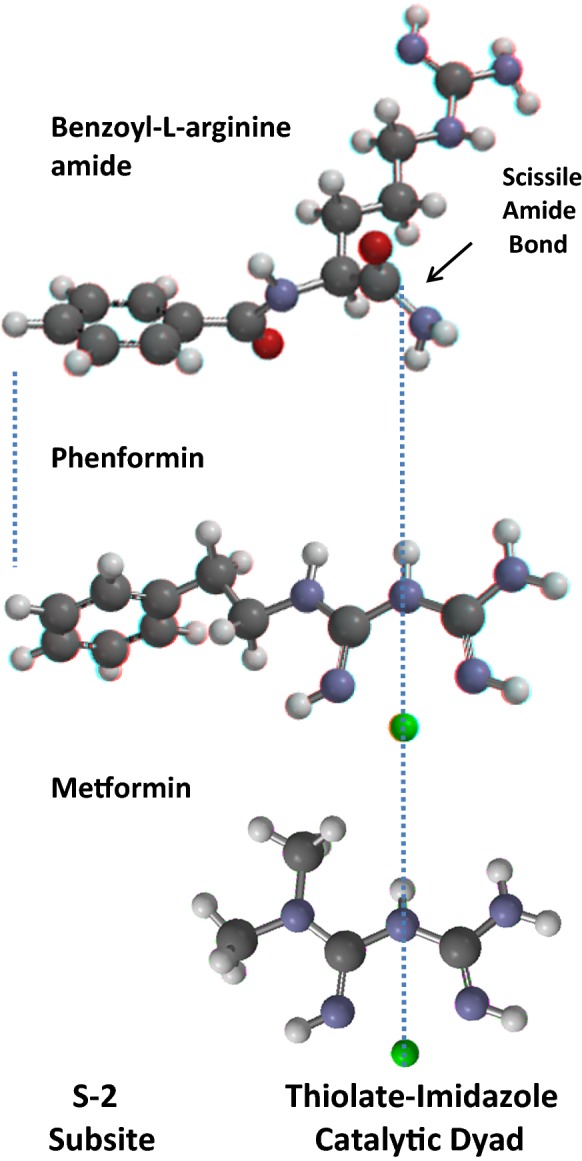
Comparison of the structures of benzoyl-l-arginine amide (BAA), and the Zn^2+^ complexes of phenformin and metformin. Structures were computed as in Fig. 4; but illustrated without the mesh. The structure of the phenformin-Zn^2+^ complex is identical to Fig. 4, but shown here from a different perspective. BAA is an artificial amide substrate of diverse proteases; the amide bond is scissile. Zn^2+^ inhibits the first step in the peptidolytic reaction. Papain-like proteases have a well-known substrate-binding site known as the S2 site (see the text). When the phenyl moiety of BAA binds to the S2 site, then the scissile amide bond is aligned with the CysHis catalytic partners and is cleaved. However, when the phenyl moiety of the phenformin-Zn^2+^ complex binds to the S2 site, then the inhibitory Zn^2+^ cation is directed to the location of the CysHis catalytic partners; and the protease is inhibited. Slight rotations or flexions of bonds can yield even closer dimensional correspondence. The Zn^2+^ complex of metformin does not have the phenyl ring; and the dimethyl moieties do not align with the S2 site when the metal aligns with the catalytic pair. Metformin inhibits cell protein degradation with a potency that is far less than metformin, i.e. ≈ 10 μM vs. ≈ 0.01 μM (Sweeney et al. 2003; Lockwood 2010, and see text). Thus, interaction of the phenyl ring with the S2 site is an important determinant of inhibitory potency. (Color figure online)

Protease reactions were originally assayed with crude substrate protein preparations, e.g. gelatin, albumin, casein or hemoglobin. In order to characterize the hydrolysis of defined single bonds, various artificial substrates were synthesized in the 1920s. Proteases were known to have activity toward ester bonds and non-peptide amides. BAA emerged from many attempted syntheses of artificial substrates (Fig. 5); the amide bond is scissile. Cleavage of the amide bond of BAA was assayed as generation of ammonia (reviewed in Taylor et al. 1974). BAA is a surrogate substrate for diverse proteases (Bergmann et al. 1939; Greenstein and Leuthardt 1946).

## The pharmacological history of biguanide and its derivatives

While biochemists were blindly screening for artificial protease substrates, medicinal chemists were blindly screening for anti-malarial drugs. These groups were unaware of the relationship that has now emerged from their separate efforts.

*An unanticipated relationship was discovered between anti-malarial biguanide derivatives and anti-diabetic biguanide derivatives*. Biguanide was synthesized long ago (Bamberger and Dieckmann 1892). Biguanide is a base, which accepts a proton and becomes charged at neutral pH. Tautomeric structures are illustrated in Bharatam et al. (2005) and Katritzky et al. (2010). Complexation of a metal cation replaces the added proton, imposing the charge of the metal (Fig. 2).

Biguanide was recognized as an anti-hyperglycemic agent in the 1920s (Slotta and Tschesche 1929), and then ignored after insulin was discovered. In the 1940s hundreds of thousands of chemicals were blindly screened for anti-malarial action. From this multitude of chemicals, various derivatives of biguanide were found to have anti-malarial action. A large number of different biguanide derivatives were synthesized and tested as described in old papers and patent applications (e.g. Curd et al. 1945; Curd and Rose 1946). Proguanil was discovered in this work (Fig. 7). Proguanil is now widely used to prevent or treat malaria in combination with atovaquone (Malarone®).

**Fig. 6:**
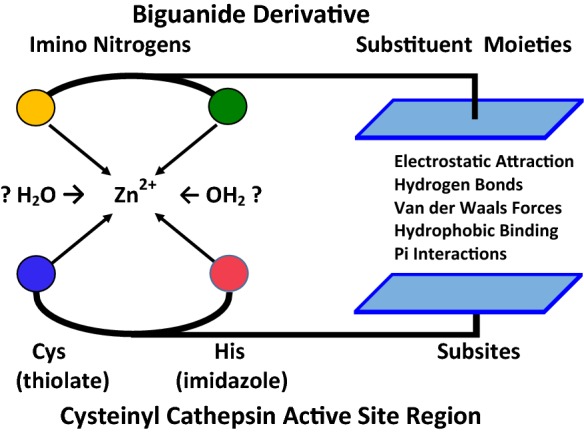
Multiple possible binding forces involved the stabilization of a “Zn^2+^ sandwich” between various biguanide derivatives and the active site regions of cysteinyl cathepsins. Left side: Zn^2+^ has 6 ligand-binding sites. Biguanide forms bidentate Zn^2+^ complexes through the two imino nitrogens (Figs. 2, 4). Catalytic partners consisting of Cys(thiolate)-His(imidazole) also have bidentate affinity for Zn^2+^. The thiolate-imidazole catalytic partners, and the imino biguanide nitrogens can reversibly form a mixed complex with a centrally coordinated Zn^2+^ i.e. a “drug-Zn^2+^-protease sandwich”. Access of water to the 2 unoccupied sites of Zn^2+^ in the recessed active site is uncertain. Right side: Diverse papain-like proteases have various arrays of substrate-binding sites on the protease surface surrounding the catalytic partners (schematized collectively by the bottom square plane; see the text). Appropriate biguanide substituent moieties (top square plane) can register with the array of substrate-binding sub-sites surrounding the metal sandwich. However, drug interactions with the protease surface surrounding the catalytic pair need not involve defined binding pockets. Many different substituents of biguanide at the 1 or 1 and 5 positions are possible (Fig. 1). The *selectivity* of the inhibitory interaction depends upon the extent of 3D structural and chemical complementarity between substituents and protease surface. The *potency* of inhibition is a different property, which depends upon attractive vs. repulsive forces stabilizing the metal sandwich. (Color figure online)

**Fig. 7:**
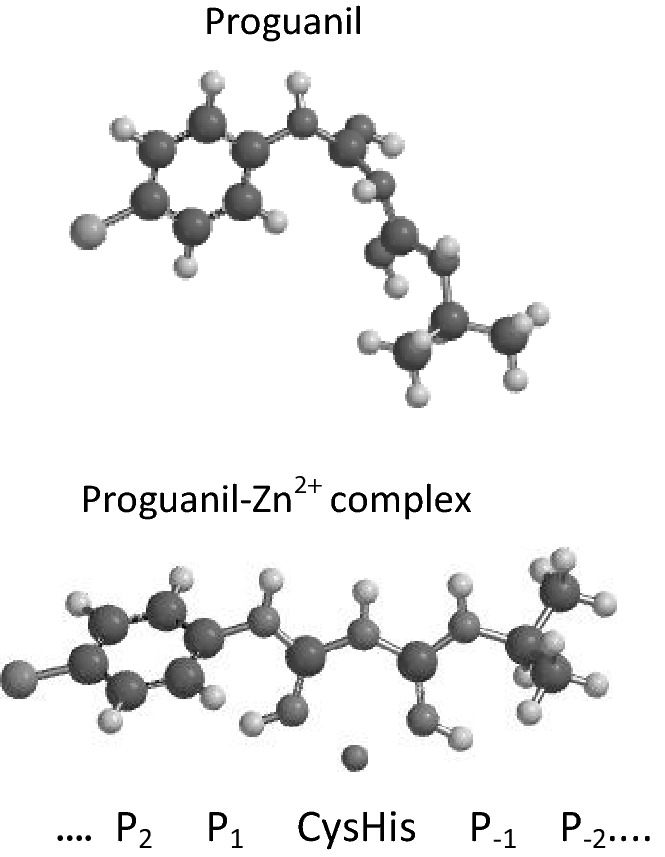
Structure of proguanil and the proguanil- Zn^2+^ complex. Structures were computed as in Fig. 4 with and without coordinated Zn^2+^. Proguanil inhibits protein degradation in bioassay (Sweeney et al. 2003). In this cartoon the correspondences between the protease binding sites and the inhibitor structure are hypothetical approximations for the sake of illustration. The proguanil-Zn^2+^ complex might interact with substrate-binding subsites flanking both sides of the catalytic partners. However, search of chemical databanks for biguanide derivatives with required inhibitory features will not be accurate unless the structures of their metal complexes are accounted for

A hypoglycemic side effect of various biguanides was noted in humans. This glucose-lowering action reawakened interest in the anti-diabetic action of biguanides. Pursuit of this side effect led to the eventual introduction of phenformin, buformin and metformin into anti-diabetic usage. Only metformin is now used due to superior safety.

*Proguanil has multiple mechanisms of action against malaria, including anti-proteolytic action*. The anti-parasitic action of proguanil was initially attributed to inhibition of dihydrofolate reductase by the metabolite cycloguanil. However, various biguanide derivatives that do not inhibit dihydrofolate reductase do have anti-malarial action. Conversely, much of the anti-malarial action of proguanil is independent of its anti-folate effect (Fidock and Wellems 1997). Thus, proguanil and other biguanides are drugs of multiple anti-malarial mechanisms. Inhibition of parasite CysCts inhibits intra-erythrocytic growth of parasites (reviewed in Sijwali and Rosenthal 2016). Parasite hemoglobinolysis results in a large accumulation of heme-Fe^3**+**^ and free Fe^3**+**^ within erythrocytes. Biguanide binds metal cations from most of the periodic table, including Fe^3**+**^ (Prugnard and Noel 1996). A sufficient concentration of Fe^3**+**^ alone can inhibit CysHis/CysHis(X) proteases; although Fe^3**+**^ is much less potent than Zn^2**+**^ (Lockwood 2006). We suggested that metformin is a metal-interactive anti-proteolytic agent, which might improve anti-malarial drug combinations (Lockwood 2010; Sharma et al. 2016b, c). As predicted, metformin has recently been found to increase the anti-malarial action of other drugs (Oriaifo 2018); multiple mechanisms have been suggested.

**Fig. 8:**
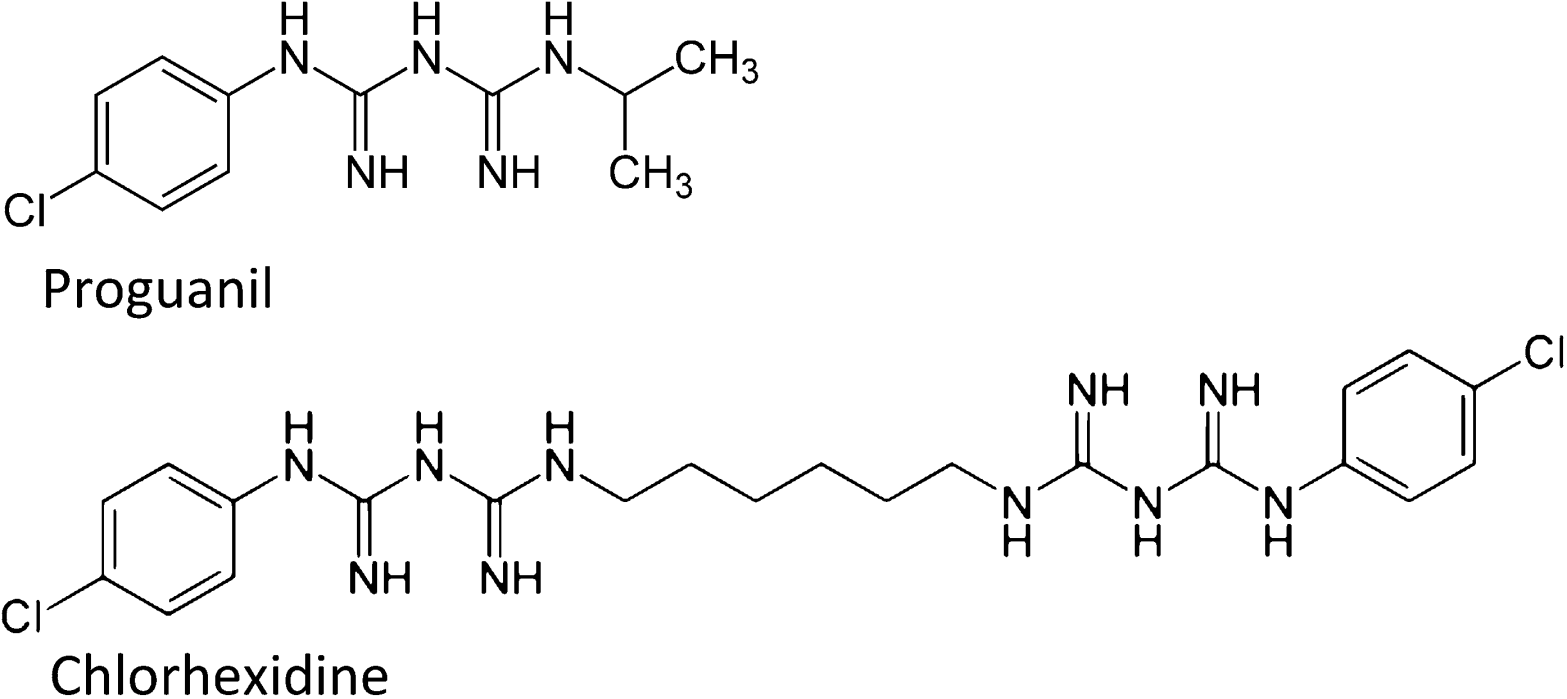
Comparison of the structures of proguanil and chlorhexidine. Chlorhexidine is widely used as an anti-bacterial agent. Chlorhexidine is a Zn^2+^-interactive inhibitor of the bacterial cysteinyl proteases known as “gingipains”; these contribute to periodontal disease. Zn^2+^ increases the anti-proteolytic potency of chlorhexidine by 3- to 30-fold (Cronan et al. 2006, and see text)

*Structural correspondences exist among phenformin, proguanil and chlorhexidine (Fig*. 8*)*. Chlorhexidine is an antibacterial agent that is widely used in dental practice. “Gingipains” are secreted bacterial cysteinyl proteases that contribute to periodontal disease. Chlorhexidine is a Zn^2**+**^ -interactive inhibitor of these proteases. Zn^2**+**^ increases chlorhexidine inhibition of gingipains by 3- to 30-fold (Cronan et al. 2006).

## Comparison of BAA and the phenformin-metal complex identifies the substrate properties of one and the inhibitory properties of the other without knowledge of the protease active site

If the structure of an enzyme active site is known, then required features of an inhibitor can be approximated with computational chemistry. Vast chemical libraries can be searched for inhibitory candidates with necessary structural and chemical attributes. However, ligand–protein modeling is hindered by uncertainties surrounding the structures of protease active sites. Ligand binding to a protein site can cause conformational changes in both participants as described by the “ligand-induced fit” principle of Koshland (1995). Modeling the interaction of a rigid ligand with a rigid binding site does not account for conformational changes in either. Chemical databanks do not store information on the effects of solvent, ions, or other molecules on the structures of ligands or binding sites as illustrated for proguanil (Fig. 7). Moreover, no present approach can account for all inter-molecular forces that determine ligand–protein interactions (discussed in Guedes et al. 2018). Indeed, some of the ligand–protein binding forces are not fully understood, and cannot be modeled, e.g. pi-electron interactions (see below section).

Substrate-inhibitor analogizing differs from ligand-protease modeling. Interpretation of the analogies between a known substrate and a known inhibitor requires no knowledge of the higher-order protease structure; although that information can be very helpful. When the phenyl rings of BAA and the phenformin-Zn^2**+**^ complex are superimposed, then the inhibitory metal is positioned at the location of the scissile bond under the conformation of the functioning protease (Figs. 4, 5).

## How is the analogy between BAA and the phenfomin-Zn^2+^ complex related to the active site structures of diverse proteases?

The active site region of papain-like proteases is located in a “groove” or “cleft” or “valley” between two lobular domains (illustrated for cathepsin B in Sweeney et al. 2003). A substrate amino acid sequence registers with the array of binding sites flanking the catalytic partners. The catalytic partners hydrolyze the peptide bond that is presented to them by the substrate binding orientation. Inhibitors can act by (a) competing with substrate amino acids for binding to subsites (competitively or non-competitively) or (b) inactivating the catalytic reaction, or (c) both. The phenformin-Zn^2**+**^ complex simultaneously interferes with both substrate binding and the catalytic mechanism (Figs. 5, 6).

*Schechter and Berger proposed that multiple binding pockets, or subsites (“S”), correspond to multiple substrate amino acid residues (“P”) in a linear relationship*. The substrate amino acid sequence was proposed to bind within the groove between domains, i.e. like “beads on a string” (Schechter and Berger 1968, and reviewed in Schechter 2005). These binding subsites correspond to the linear sequence of substrate amino acids on either side of the scissile bond.

$${\text{S}}_{\text{n}} \ldots {\text{ S}}_{ 3} {\text{S}}_{ 2} {\text{S}}_{ 1} {\text{CysHis}}\left( {\text{X}} \right){\text{S}}^{\prime}_{ - 1} {\text{S}}^{\prime}_{ - 2} {\text{S}}^{\prime}_{ - 3} \ldots {\text{S}}^{\prime}_{{ - {\text{n}}}}$$

Substrate binding affinity and orientation was proposed to result from combined interactions of multiple S–P interactions on one or both sides of the catalytic partners. Inhibitors were proposed to compete with substrate peptides for binding to various S and/or S′ subsites. The “P” sites indicate the amino terminus of substrate peptides; and the P′ or “P prime” sites indicate the carboxyl terminus. The asterisk indicates the scissile bond:

$${\text{H}}_{ 2} {\text{N }} - {\text{ P}}_{\text{n}} \ldots {\text{P}}_{ 3} {\text{P}}_{ 2} {\text{P}}_{ 1} *{\text{P}}^{\prime}_{ - 1} {\text{P}}^{\prime}_{ - 2} {\text{P}}^{\prime}_{ - 3} \ldots {\text{P}}^{\prime}_{{ - {\text{n}}}} - {\text{ COOH}}$$

The Schechter–Berger concept has been debated for a half-century. The exact positions, dimensions, spacing, borders, overlap and substrate interactivity of the sub-sites is ultimately speculative. It is difficult to distinguish a binding pocket that was selected for advantage, from a random constellation of amino acids on a protease surface. Interpreting substrate amino acid sequences as “beads on a string” ignores interactions between the 3-D structures of proteases and substrates or inhibitors. Bond rotations or flexions can alter the S–P complementarity of substrates and inhibitors. In most papain-like proteases the substrate binding is not highly specific insofar as the subsites can accept variable combinations of substrate amino acids. A peptide substrate or an inhibitor can be bound in various orientations. The “other face” of a bound substrate is exposed to non-selective solvent attraction or hydrophobic exclusion. The Schechter–Berger concept is now considered to be an oversimplification of the interactions of proteases with peptides and inhibitors; however, it remains useful for some purposes.

*A puzzling substrate-binding interaction is shared by nearly all papain-family proteases*. One feature of the Schechter–Berger concept has proved valid over many years of scrutiny. Long ago they reported: “…in papain one of the subsites of the active site - namely S2 - specifically interacts with phenylalanine residues…” (Schechter and Berger 1968). The S2 subsite position has since been confirmed as a major determinant of substrate preference and reaction kinetics in many proteases (Turk et al. 2012; Corvo et al. 2018). Nearly all proteases of the papain superfamily appear to have an S2 position that accepts bulky hydrophobic amino acids, e.g. the phenyl moiety of BAA. Some of the caspases, calpains and serine proteases also seem to have an influential S2 substrate-binding position (Campbell and Davies 2012; Ono et al. 2016; Paireder et al. 2016).

The S2P2 interaction is unexplained and puzzling. The constellation of amino acids comprising the S2 subsite is not the same in all papain-like proteases. The sizes and boundaries of various S2 sites are not precisely defined. The S2 subsite position can accept dissimilar substrate side groups other than phenylalanine. Non-specific hydrophobic exclusion from solvent to a “greasy spot” on a protein surface can provide a strong, non-selective binding force. Nonetheless, CysCts have diverged and converged throughout evolution while the influence of the S2 position has been conserved in many. The mystery of the S2 site might be somehow related to the pi electron system of the phenyl ring (see below).

*Metformin indicates that a phenyl ring at the P2 position is unnecessary for Zn*^2**+**^*-interactive inhibition of CysCts; however, the S2P2 interaction is a major determinant of potency*. Both metformin and phenformin are Zn^2**+**^-interactive inhibitors of cell protein degradation as observed in viable tissue bioassay (Lockwood 2010). These drugs share the same biguanide pharmacophore; however, the dimethyl moieties of metformin are not positioned at the S2 position when the complexed metal is located between thiolate and imidazole partners (Fig. 5). The required anti-proteolytic concentration of metformin in tissue bioassay (≈ 10 μM) is approximately 1000 times greater than phenformin (≈ 0.01 μM). Although the anti-proteolytic potency of metformin is weak; it is effective at the therapeutic concentration employed (≈ 25 μM). Supra-therapeutic metformin concentrations have been reported to have many other experimental actions. This reviewer has found no other proposed mechanism of metformin action that has been verified at 10 μM concentration.

Ligand–protein modeling of metformin binding to the active site of cathepsin B indicates that there is no single binding mode. Dimethyl groups of the metformin-metal complex can bind to the active site in many possible orientations that are associated with metal sharing between thiolate–imidazole partners (unpublished observation). There is no necessary “S-P” correspondence when the complexed metal is aligned with the catalytic pair. The two methyl groups of metformin impart a partial hydrophobic character to complexed Zn^2**+**^. Solvent exclusion can promote non-specific hydrophobic binding of the metformin-metal complex to the protease surface at multiple positions immediately surrounding the catalytic pair. With the metal shared between thiolate and imidazole, the dimethyl moieties of metformin can pivot to multiple binding sites on the surrounding protease surface without involvement of the S2P2 interaction. In several binding orientations, amide protons of metformin can also interact with the electronegativity of carbonyl oxygens of the peptide backbone (illustrated for phenformin in Sweeney et al. 2003). Nonetheless, the 1000-fold difference in potency of phenformin and metformin suggests that the S2P2 interaction is an important determinant of binding affinity and anti-proteolytic action.

## Interaction of Zn^2+^ and Zn^2+^ -interactive drugs with the sulfhydryl/thiolate-imidazole/imidazolium reaction mechanism under in vivo and ex vivo reaction conditions

Peptidase, esterase and non-peptide amidase reaction mechanisms of proteases differ (e.g. Whitaker and Bender 1965 and others); however, details are ultimately speculative. Zn^2**+**^ insertion between thiolate and imidazole stops peptidase, esterase and non-peptide amidase reaction mechanisms. In the absence of metals the CysHis/CysHis(X) peptidolytic reaction involves formation of thiolate anion and imidazolium cation by transfer of the proton (illustrated in Berg et al. 2002). A lone electron pair of the nearby imidazole ring attracts the proton from the cysteine sulfhydryl. The proton attraction of imidazole lowers the pH at which the sulfhydryl ionizes so as to become a nucleophile. Loss of the proton from the sulfhydryl transforms it into a nucleophile. This enables nucleophilic attack of the thiolate anion on the carbonyl carbon of the peptide bond.

*A thiolate anion neighboring an imidazole ring exhibits selective affinity for Zn*^2**+**^*in preference to other endogenous metal species (Kochanczyk et al.*^2015^*; Kluska et al.*^2018^). Zn^2**+**^ interaction with the catalytic pair involves factors in addition to the charge of the metal cation. For example, the affinity and inhibitory potency of Zn^2**+**^ is greater than Fe^3**+**^. However, the affinity and inhibitory potency of Zn^2**+**^ is far less than its group IIB cousins, Cd^2**+**^ and Hg^2**+**^, by orders of magnitude. The high affinities of Cd^2**+**^ and Hg^2**+**^ suffice to titrate the concentration of CysHis active sites with a nearly 1:1 stoichiometric equivalence.

There is no absolute value for the potency of Zn^2**+**^ inhibition of various CysCts in vivo or ex vivo. The extent of metal inhibition is strongly dependent upon combined reaction conditions. With purified cathepsin B the 50% inhibitory concentration of Zn^2**+**^ is approximately 1 μM under 5 mM DTT and pH 5.5. Firstly, the inhibitory action of Zn^2**+**^ is opposed by the stimulatory action of reducing factors, including DTT (Balce et al. 2014, 2016). The dithiols of DTT bind and buffer Zn^2**+**^. Thus, the actual free Zn^2**+**^ intensity in a typical protease assay is less than that added experimentally. Secondly, protons compete with metal cations for binding sites on proteins; therefore metal inhibition of CysCts is also pH dependent. In general, Zn^2**+**^ inhibition in cysteinyl protease assays is much greater at higher pH and lesser DTT concentration.

*The anti-proteolytic synergy between biguanides and Zn*^2**+**^*in living tissue is greater than the synergy measured in protease assay*. The perfused tissue bioassay provides a more reliable indication of the anti-proteolytic potency of various biguanide derivatives than standard CysCt protease assay. The bioassay accounts for endogenous concentrations all participants influencing formation of the drug-Zn^2**+**^-protease sandwich. Exposure to phenformin (0.01 µM) does not require experimental addition of extracellular Zn^2**+**^ for the most rapid time course of its inhibitory action. Due to its great potency, phenformin can acquire sufficient intracellular Zn^2**+**^ for maximal anti-proteolytic action at 0.01 µM drug concentration. In contrast, the anti-proteolytic action of therapeutic metformin concentration (10 µM) was delayed and submaximal in the absence of extracellular Zn^2**+**^ added to the perfusate. Exposure to physiological extracellular Zn^2**+**^ concentration and therapeutic metformin concentration were highly synergistic. These experimental results are expected of two agents with similar metal-interactive mechanisms of action, but a great difference in binding affinity for the CysCt active site.

We reported that metformin adds little to the inhibitory action of Zn^2**+**^ toward protease assays as observed under standard assay conditions of 5 mM DTT at pH 5.5 (details described in Lockwood 2010). In contrast, phenformin and Zn^2**+**^ were synergistic under identical assay conditions. This difference among biguanide derivatives is probably due to lesser binding affinity of the metformin-Zn^2**+**^ complex for the protease in conjunction with the competition of DTT for Zn^2**+**^ binding. We later found that the inhibitory synergy between metformin and Zn^2**+**^ in protease assay is appreciably greater at higher pH with less competing DTT concentration (unpublished observation). Therefore, therapeutic metformin concentration does cause Zn^2**+**^-interactive inhibition in protease assay; however, its potency is much less than phenformin.

Zn^2**+**^ naturally influences many cellular processes and enzyme reactions, e.g. some of the kinases. It is conceivable that biguanides might inhibit lysosomal proteolysis indirectly by interference in vacuolar/cellular regulation of metals or pH, or autophagic substrate accumulation. Moreover, an agent that modifies the interaction of metals with biomolecules might have multiple effects that are unrelated to protein degradation (Wu et al. 2017); additional actions are not ruled out.

## The stabilities of various drug-metal-protease complexes are the combined result of the binding of biguanide substituent moieties to protease subsites, and the mutual affinities of biguanide and catalytic partners for a central metal cation (Fig. 6)

Simultaneous Zn^2**+**^ interactions with biguanide and protease can increase the fraction of time that the catalytic partners are occupied by Zn^2**+**^, and competitively decrease effective collisions of substrates with binding sites (Fig. 6). The binding force between the thiolate–imidazole pair of the protease and the two imino nitrogens of biguanide is their mutual affinity for the particular metal cation that is coordinated between them. Multiple binding forces can attract biguanide substituent moieties to the protease surface surrounding the catalytic partners; these include hydrogen bonds, electrostatic charge attractions, Van der Waals short-range forces, pi electron interactions, and solvent exclusion to “greasy spots” on a protein surface, i.e. hydrophobic binding.

In the absence of metals, phenformin is a competitive inhibitor of substrate hydrolysis by cathepsin B in protease assay (Sweeney et al. 2003). Substrate binding to the protease surface does not compete with inhibitory Zn^2**+**^ binding to the catalytic partners. Inhibition of substrate hydrolysis by the phenformin-Zn^2**+**^ complex theoretically involves mixed kinetics that cannot be described by customary Michaelis–Menten kinetics.

*Recent speculations as to the pi system of aromatic rings are of particular interest in relation to the interaction of the phenylalanine with the S2 site*. Theoretical advances suggest that pi electron systems can interact with cations, anions, other pi systems, and solvent (Meyer et al. 2003; Schottel et al. 2008; Dougherty 2012; Makwana and Mahalakshmi 2015). The influence of pi electrons on inter-molecular interactions is an old topic that is now taking on new significance. Phenylalanine interactions at the S2 site could involve uncharacterized pi interactions as well as hydrophobic solvent exclusion and other binding forces. This might account for the confusing versatility of S2P2 substrate acceptance as described above. However, such speculation is largely theoretical with little experimental basis.

*Non-biological metals can form a stable biguanide association that can be administered as a preformed complex*. The binding forces and “on–off” kinetics depend, in part, upon the identity of the particular metal in the hetero-complex. For example, the preformed ^99m^Technetium- phenformin complex is sufficiently stable to be used in radiopharmaceutical imaging after parenteral administration (Fuks et al. 2011). Some of the non-biological metal species might be relevant to medicinal or other applications of protease inhibition, e.g. platinum, vanadium, chromium, iridium, paladium, nickel, technetium, lanthanum, cesium, yttrium, samarium, and others (Woo et al. 1999; Patrinoiu et al. 2003; Fuks et al. 2011; Al-Saif and Refat 2013; Badea et al. 2013; Beygzadeh et al. 2013; Shahabadi and Heidari 2014; Refat et al. 2015; Mahmoud et al. 2016; Chen et al. 2018; Mihalache et al. 2018). Metal toxicity is an obvious concern.

*Inhibitor specificity and potency are determined by different interactions with the protease active site*. With the same metal, the *specificity* of a biguanide inhibitor depends upon its unique complementarity with the array of protease binding sites. If the active site structures of multiple proteases are almost identical, then a single inhibitor cannot discriminate between them. Accordingly, some biguanide derivatives might coordinately inhibit groups of CysCts, which share common structural features (e.g. Vizovišek et al. 2015). Inhibitory potency depends upon the combined affinity of metal and substituents for the active site. A hypothetical biguanide derivative that uniquely complements the binding sites of a particular protease can have a high degree of inhibitory selectivity, but low affinity and potency. A biguanide-metal complex with low affinity can selectively occupy a specific active site 100% of the time if the (non-toxic) drug concentration is adequate.

## Does phenformin reveal features of convertase inhibitors?

Long ago it was reported that various phenylalanine-containing structures competitively inhibit mammalian cathepsin C (Fruton and Mycek 1956). Mammalian cathepsin C has dipeptidyl amino peptidase (DPAP) activity of particular interest here. Cathepsin C serves in general protein turnover in most cell types (Bullón et al. 2018). Cathepsin C also serves a specialized role in cells mediating the inflammatory response. The complex structure and biology of cathepsin C are beyond the present scope (see Korkmaz et al. 2018; Seren et al. 2018); however, some of its features are relevant to “polytherapeutic” treatment of inflammatory conditions and perhaps parasite infections.

Cathepsin C is a convertase, which activates the zymogens of multiple serine proteases in multiple cell types. Cathepsin C is naturally inhibited by the endogenous peptide, cystatin F (Kos et al. 2018; Liang et al. 2016). This protease is present in the exocytic granules of several differentiated inflammatory cell types (Pham and Ley 1999). Cathepsin C activates pro-inflammatory serine protease zymogens in these exocytic granules; e.g. granzyme A and B, elastase and cathepsin G in neutrophils, or chymase and tryptase in mast cells. These activated serine proteases are released into the extracellular space where they are fundamental to the normal and excessive inflammatory responses. Cytotoxic lymphocytes from CatC (−/−) knockout mice have normal amounts of granzyme A and B zymogens, but these proteases retain their pro-domains and remain inactive in the absence of cathepsin C (Pham and Ley 1999).

Extracellular serine proteases from various inflammatory cells are involved in diverse diseases, including rheumatoid arthritis, inflammatory bowel disease, asthma, chronic obstructive pulmonary disease, sepsis, cystic fibrosis and others. In theory, “whole body” inhibitors of cathepsin C can decrease the extracellular action of multiple pro-inflammatory serine proteases that are released by specialized inflammatory cell types (Guarino et al. 2017). This implies that targeting cathepsin C can have pleiotropic “bioamplified” actions against inflammation and autoimmunity (Méthot et al. 2007). Major drug companies are currently seeking cathepsin C inhibitors.

It has been reported that the fluorogenic derivative of the Phe-Arg dipeptide substrate, Benzyloxycarbonyl-phenylalanylarginine-4-methylcoumaryl-7-amide, (CBZ-Phe-Arg-AMC) inhibits the DPAP reaction toward other peptide derivatives (Wang et al. 2011). Parasite DPAPs are candidates for anti-malarial targeting also (Deu 2017; Deu et al. 2018; Lehmann et al. 2018). It would be interesting to determine whether mammalian or parasite DPAPS might be selectively sensitive to biguanide agents with structural analogies to BAA and the phenformin-Zn^2+^ complex.

Cathepsin C is not the only catheptic convertase. Cathepsin B has exo-dipeptidase activity as well as endo-protease activity. Cathepsin B also serves to activate pro-forms of various other proteases. Dysregulated cathepsin B is implicated in pathogenic activation of trypsinogen within the pancreas before release into the digestive tract; this results in pancreatitis (reviewed and discussed in Sendler et al. 2018).

## Summary and future possibilities

Various methods to synthesize biguanides have been reported (Curd et al. 1945; Mayer et al. 2004; LeBel et al. 2005; Katritzky et al. 2010; Abbas et al. 2016, and others). The biguanide pharmacophore has low toxicity; however, the risk of each derivative must be determined. Some larger biguanide derivatives can create self-assembling metallo-supramolecules (e.g. McMorran et al. 2013; McKay et al. 2017); these are presumably toxic. The registry and affinity of various derivative moieties with multiple sub-sites can create a range of specificities and potencies against diverse proteases. Coordinate inhibition of multiple CysCts and lysosomal function might be of value for some purposes. The benefits of stable, preformed biguanide complexes with non-biological metals might exceed the risks where complete inhibition for a short time period is required, e.g. viral or parasite infections. Anti-neoplastic applications are an open question.

The structure of phenformin is a model for the appropriate relationship between the phenyl ligand at the S2 site and the complexed Zn^2**+**^ cation at the catalytic pair of many papain-like proteases (Figs. 5, 6). Inhibitor binding at other subsites presumably depends upon the individual structures of diverse proteases. In tissue bioassay, the 1000-fold difference in anti-proteolytic potency between metformin and phenformin indicates that the S2P2 correspondence is an important determinant of the binding interaction of rate-limiting proteases. However, metformin illustrates that S2P2 structural correspondence is not essential for Zn^2**+**^-interactive inhibition of protein degradation with lesser potency.

Finally, cellular Zn^2**+**^ regulation influences multiple proteolytic processes by mechanisms in addition to the CysHis catalytic mechanism. Other metallophores or other metal-related anti-proteolytic interventions might be discovered, e.g. allosteric modulators. However, present knowledge of metal-interactive inhibition of peptidolysis with biguanide derivatives enables forseeable discovery of many new drugs against individual CysHis(X) proteases or groups of them.

## Abbreviations

CysCts: Cysteinyl cathepsins
CysHis: Cysteine-histidine catalytic dyad
CysHis(X): Cysteine-histidine (X amino acid) catalytic triad
DPAP: Dipeptidyl amino peptidase
BAA: Benzoyl-l-arginine amide

## IUPAC names

Guanidine: Guanidine
Biguanide: Guanyl guanidine or 1-(diaminomethylidene)guanidine
Phenformin: 1-(Diaminomethylidene)-2-(2-phenylethyl)guanidine
Metformin: 3-(Diaminomethylidene)-1,1-dimethylguanidine
Proguanil: (1E)-1-[Amino-(4-chloroanilino)methylidene]-2-propan-2-ylguanidine
Benzoyl-l-arginine amide: N-[(2S)-1-amino-5-(diaminomethylideneamino)-1-oxopentan-2-yl]benzamide
